# Advances in non-coding RNA profiling for neurological diseases

**DOI:** 10.3389/fgene.2013.00005

**Published:** 2013-01-25

**Authors:** Murray J. Cairns, Jannet Kocerha

**Affiliations:** ^1^School of Biomedical Sciences and Pharmacy, and Hunter Medical Research Institute, University of NewcastleCallaghan, NSW, Australia; ^2^Schizophrenia Research InstituteSydney, NSW, Australia; ^3^Division of Neuropharmacology and Neurologic Disease, Yerkes National Primate Research CenterAtlanta, GA, USA; ^4^Department of Human Genetics, Emory University School of MedicineAtlanta, GA, USA

Over the past decade, the classifications and volume of non-coding RNA (ncRNA) transcripts have grown profoundly, spurred by transcriptomic sequencing efforts (Bartel, 2009; Guttman et al., 2009; Djebali et al., 2012). This has identified an array of human disorders associated with some degree of ncRNA dysregulation (Taft et al., 2010), prompting an expanding need to characterize ncRNA expression in targeted tissues. Brain disorders, including neurodegenerative and psychiatric disease, have increasingly been the subject of ncRNA profiling studies (Kocerha et al., 2009a,b; Beveridge et al., 2010; Im and Kenny, 2012). The technology to probe for ncRNA expression, however, varies, depending on the class of transcripts to be examined. Arguably, to date, the resources to assess microRNA (miRNA) expression are the most comprehensive, spanning from classic hybridization arrays to real-time PCR arrays. However, with the discovery of long ncRNAs (lncRNAs) in the brain, such as natural antisense transcripts (NATs) and large intergenic non-coding (Linc) RNAs (Guttman et al., 2009; Modarresi et al., 2012), the access to new technology for examination of those transcripts is also expanding.

One of the challenges with the emerging field of ncRNAs is the frequent updates to the current database of annotated transcripts and occasional changes in nomenclature, making it essential to keep current with available sequence information. For example, the miRBase server, which contains all annotated miRNAs, has already been updated through 19 different versions to date. Importantly, these updates can include addition or deletion of transcripts due to various reasons. Technology suppliers usually lag behind databases with their ncRNA platforms, although some have the capacity to include custom content if required for a particular study. The fluctuation in transcript annotation is also important to consider in the context of lncRNAs, as fixed platforms take longer to accommodate changes in this rapidly emerging genomic dimension. For this reason, RNA sequencing (RNA-Seq) approaches that don't rely on *a priori* information and annotation have the advantage of flexibility. This flexibility, however, comes at some expense in terms of accessibility, analysis, and quantification.

Despite considerable advances in ncRNA analysis, there remains technical challenges and key differences between platforms and approaches that may add further variation to an area already subject to heterogeneity. For example, postmortem miRNA profiling in schizophrenia has revealed significant changes associated with the disorder (Perkins et al., 2007; Beveridge et al., 2008, 2010; Santarelli et al., 2011). While compelling, the correspondence between different laboratories has not been as high as expected (Beveridge and Cairns, 2012). This could be a feature of disease heterogeneity or the dynamics of miRNA function and distribution in the brain, or a consequence of variation in cytoarchitecture and cellular composition. Some of these parameters will be controlled better in larger cohorts, and by screening multiple sub-regions or through more precise sampling. Other sources of variation and artifact may reside at the level of molecular analyses, including technical variation between various platforms and data analysis techniques, whether they be hybridization, amplification, or sequencing based. All of these approaches will have some bias and make some assumptions based on the normalization strategy. For example, all three of these approaches usually entail some degree of amplification, which is notorious for introducing bias. The extent of amplification will depend both on the sensitivity of the instrumentation and the amount of RNA that can be devoted to ncRNA analysis. As the cellular specificity is increased, the need for further amplification or pre-amplification also increases. Pre-amplification may result in detection of non-neuronal ncRNAs in the brain tissues analyzed, therefore, accurate interpretation of the results is critical to avoid the over interpretation of contaminating template. There is also other bias introduced by methodologies such as RNA-Seq that require ligation of linkers for cDNA library construction. Even the type of RNA extraction can influence results, particularly with small RNAs that have large sequence dependent differences in physicochemical properties that affect their affinity for purification matrices or capacity for precipitation. For example, the standard Trizol preparation of small RNA has been shown to be influenced by the magnesium concentration (Kim et al., 2012). ncRNA expression may also vary significantly between distinct brain regions, either with or without the presence of neurological pathogenesis. Again, these differences can only be appreciated by more widespread collection and analysis of disease associated tissues.

RNA-Seq is contributing to significant discoveries in neuroscience research. One of the key advantages of this approach is that poorly characterized RNA variants, particularly of the non-coding variety that lack comprehensive annotation, are now captured and can be analyzed. Previously, these molecules were only seen at great expense on genome tiling arrays and a few custom non-coding arrays. This technology has the potential to make a tremendous impact in the ncRNA world as they emerge for the first time with an equal billing as the coding transcripts. This technology, however, is also subject to some limitations that need to be considered for experimental design, in addition to the issue of ligation, amplification, and normalization bias mentioned already. Firstly, there are no one-size-fits all strategy for ncRNAs. Small RNAs, such as miRNA, need to be fractionated and treated separately to their longer counterparts. Standard RNA-Seq, also poly-A selection during library preparation and as such, will not enrich for RNA transcribed by pol III promoters and other intergenic promoters. Total RNA-Seq requires a ribosomal RNA depletion step, and to fully appreciate the influence of NATs it may be necessary to use strand-specific RNA-seq. Even more selection may be necessary to find rare non-coding transcripts. For example, ncRNAs were recently found in “gene deserts” after targeted enrichment using tiling arrays (Mercer et al., 2011). With more small RNA analysis employing sequencing, depth is also important for identifying miRNA variants (isomiRs) and other variants produced through RNA editing (Peng et al., 2012). Overall, a detailed comparison of ncRNA sequences in control vs. diseased patients may open the door to identifying new transcriptomic mechanisms provoked through distinct human genetic anomalies. These advances in RNA technology and analysis hold great promise for the discovery of new ncRNA biomarkers and therapeutics for brain disorders.
